# Smoking and multiple sclerosis risk: a Mendelian randomization study

**DOI:** 10.1007/s00415-020-09980-4

**Published:** 2020-06-11

**Authors:** Marijne Vandebergh, An Goris

**Affiliations:** 1 Department of Neurosciences, Laboratory for Neuroimmunology, KU Leuven, Herestraat 49 bus 1022, 3000 Leuven, Belgium; 2 Leuven Brain Institute, KU Leuven, Leuven, Belgium

**Keywords:** Multiple sclerosis, Susceptibility, Environment, Genetics, Mendelian randomization

## Abstract

**Background:**

Striking changes in the demographic pattern of multiple sclerosis (MS) strongly indicate an influence of modifiable exposures, which lend themselves well to intervention. It is important to pinpoint which of the many environmental, lifestyle, and sociodemographic changes that have occurred over the past decades, such as higher smoking and obesity rates, are responsible. Mendelian randomization (MR) is an elegant tool to overcome limitations inherent to observational studies and leverage human genetics to inform prevention strategies in MS.

**Methods:**

We use genetic variants from the largest genome-wide association study for smoking phenotypes (initiation: *N* = 378, heaviness: *N* = 55, lifetime smoking: *N* = 126) and body mass index (BMI, *N* = 656) and apply these as instrumental variables in a two-sample MR analysis to the most recent meta-analysis for MS. We adjust for the genetic correlation between smoking and BMI in a multivariable MR.

**Results:**

In univariable and multivariable MR, smoking does not have an effect on MS risk nor explains part of the association between BMI and MS risk. In contrast, in both analyses each standard deviation increase in BMI, corresponding to roughly 5 kg/m^2^ units, confers a 30% increase in MS risk.

**Conclusion:**

Despite observational studies repeatedly reporting an association between smoking and increased risk for MS, MR analyses on smoking phenotypes and MS risk could not confirm a causal relationship. This is in contrast with BMI, where observational studies and MR agree on a causal contribution. The reasons for the discrepancy between observational studies and our MR study concerning smoking and MS require further investigation.

**Electronic supplementary material:**

The online version of this article (10.1007/s00415-020-09980-4) contains supplementary material, which is available to authorized users.

## Introduction

Multiple sclerosis (MS) is an autoimmune disease of the central nervous system, with both genetic and environmental factors implicated in its etiology [1]. Genome-wide association studies (GWASs) have identified more than 200 independent associations mediating disease risk [2–6], but these genetic factors explain < 50% of variance in MS risk between individuals [3].

Environmental risk factors associated with MS in observational studies include Epstein-Barr virus infection, low serum vitamin D [25(OH)D3], obesity and smoking [7]. Unlike genetic risk factors, environmental and lifestyle factors can be modified, with potential for prevention. Behavioural, socioeconomic, and physiological factors are strongly interrelated, which can lead to residual confounding in observational studies [8, 9]. Furthermore, causal inference from observational studies is impeded by reverse causation, selection and recall bias. To overcome this, a Mendelian randomization (MR) analysis uses genetic variants that are a proxy for environmentally modifiable exposures as an instrumental variable (IV) to assess the presence of a causal relationship between environmental factors and an outcome [10]. For three of the environmental risk factors suggested by observational studies (low serum vitamin D, obesity and smoking), sufficiently strong IVs for use in MR studies are now available. Such analyses have repeatedly supported a causal association of low serum 25(OH)D3 and obesity with increased MS susceptibility [11–15]. However, a causal relationship between smoking and MS risk remains underinvestigated.

In this study, we make use of the largest GWAS summary statistics for smoking to date [16] and apply a two-sample MR analysis to the most recent meta-analysis GWAS summary statistics for MS from the International Multiple Sclerosis Genetics Consortium (IMSGC) [3].

## Methods

### Genetic datasets

Genome-wide significant (*p* < 5 × 10^−8^) genetic variants associated with smoking initiation, a binary phenotype indicating whether an individual has ever smoked regularly, were obtained from Supplementary Table 4 of the GWAS and Sequencing Consortium of Alcohol and Nicotine use (GSCAN) study for smoking initiation [16], with the largest sample size to date involving 1,232,091 individuals. The 378 single nucleotide polymorphisms (SNPs) explain 2.3% of the phenotypic variation in smoking initiation [16]. As secondary phenotypes, we included two measures reflecting a dose- and time-effect for smoking. A total of *N* = 55 genetic variants associated with smoking heaviness, measured by cigarettes per day in 337,334 individuals, were obtained from the same study, and explain ~ 1% of phenotypic variation [16]. For other variants than those reaching genome-wide significance, full summary statistics for smoking phenotypes were downloaded (Supplementary Table 1), but are only available for up to 632,807 individuals, excluding 23andMe participants. An IV reflecting lifetime smoking has been constructed in 462,690 individuals of European ancestry from the UK Biobank by integrating information on smoking status (current, former, never), age at initiation, age at cessation and number of cigarettes smoked per day. This IV is based on 126 independent, genome-wide significant SNPs and captures 0.36% of the variance [17].

Data from The Genetic Investigation of Anthropometric Traits (GIANT) Consortium meta-analysis for body mass index (BMI) in 681,275 individuals [18] (Supplementary Table 1) were used for investigating the causality between smoking phenotypes and BMI and for correcting the effect of smoking-associated SNPs on BMI and vice versa. All primary and secondary genome-wide significant SNPs (*N* = 941) explain 6% of variance in BMI in a cohort of 8852 individuals [18]. We include the *N* = 656 primary associations listed in the study as IVs. Corresponding effects of the smoking- and BMI-associated SNPs on MS susceptibility were derived from the discovery cohorts of the latest IMSGC meta-analysis, including up to 41,505 participants (14,802 MS, 26,703 controls) [3]. For the reverse analysis of genetically predicted MS risk on smoking initiation, cigarettes per day and lifetime smoking, *N* = 138 primary SNP associations with MS were derived from Supplementary Table 7 of the latest IMSGC meta-analysis for multiple sclerosis risk [3].

### Selection of instrumental variables

Clumping and data harmonization were implemented in R v3.6.1 using the TwoSampleMR package (v0.5.1) [19]. For each genetic variant, alleles were aligned and matched so that their effects correspond to an increase in the corresponding exposure to which they are associated. The odds ratios (ORs) and *p* values of the summary statistics from the IMSGC were transformed into *β* coefficients and standard errors for subsequent analyses. SNPs with OR of exactly one were excluded. As the BMI phenotype in the GIANT meta-analysis was normalized, *β* coefficients correspond to standard deviations (SD) of BMI, with one SD equaling a mean of 4.70 BMI units (kg/m^2^) among cohorts in the GIANT consortium [18]. For smoking, the scale of *β* is on the unit of the SD of the phenotype, with a one SD increase in genetically predicted smoking initiation and heaviness corresponding to a 10% increased risk of smoking and three additional daily cigarettes, respectively [16]. Individuals who have never smoked and have no smoking exposure have a lifetime smoking score of zero, and a one SD increase is equivalent to an individual smoking 20 cigarettes a day for 15 years and stopping 17 years ago or an individual smoking 60 cigarettes a day for 13 years and stopping 22 years ago [17]. To prevent result bias by strongly correlated SNPs, SNPs were excluded from analyses if their measured linkage disequilibrium (LD) is *r*^2^ > 0.05 in the European samples of 1000 Genomes. For SNPs in LD with *r*^2^ > 0.05, the SNP with the lowest *p* value is retained. Furthermore, to prevent strand ambiguity issues and as minor allele frequencies were not available for all summary statistics, only non-palindromic SNPs were retained. For exposure-associated variants not directly ascertained in the outcome datasets in the univariable analysis, we looked for proxy SNPs in high linkage disequilibrium (*r*^2^ > 0.9) using LDlinkR package in R (v3.6.1)*.* Supplementary Table 2 contains an overview of the number of included and excluded SNPs.

In the univariable MR analysis for the effect of exposures on MS risk, a total of *N* = 297 SNPs, *N* = 38 SNPs, *N* = 111 SNPs and *N* = 529 SNPs were included for smoking initiation (Supplementary Table 3), cigarettes per day (Supplementary Table 4), lifetime smoking (Supplementary Table 5) and BMI (Supplementary Table 6), respectively. In the multivariable MR analysis, *N* = 576 SNPs associated with either smoking initiation or BMI (Supplementary Table 7) were retained. To investigate the effect of BMI on smoking initiation and the reverse, *N* = 530 SNPs (Supplementary Table 8) and *N* = 266 SNPs (Supplementary Table 9) were included, respectively. Finally, to examine causality between BMI and cigarettes per day, *N* = 531 SNPs were used (Supplementary Table 10), and for the reverse analysis *N* = 29 SNPs (Supplementary Table 11).

For the reverse analysis of genetically predicted MS risk on smoking initiation, cigarettes per day and lifetime smoking, a total of *N* = 118 SNPs were included (Supplementary Table 12, 13 and 14, respectively).

### Statistical analyses

MR analyses were implemented in R v3.6.1 using the TwoSampleMR package (v0.5.1) [19].

Effects of IVs on outcomes were estimated using the traditional MR approach, that is a multiplicative random-effects inverse-variance weighted (IVW) analysis [19, 20]. Sensitivity tests more robust to potential pleiotropy but potentially less powered included MR Egger [21], weighted median regression [22] and the mode-based estimator (simple and weighted) [23]. We considered as MR results suggestive of causal effects those that were concordant in direction across multiple MR approaches and pass nominal significance in IVW MR.

The Cochran *Q* test and *I*^2^ statistic [24] were calculated to measure the degree of heterogeneity across the individual effect estimates derived from each genetic variant [25]. Horizontal pleiotropy was evaluated based on the intercept obtained from the MR Egger analysis being significantly different from zero [21, 26] and by visual inspection of the funnel plot, where asymmetry is indicative of horizontal pleiotropy [19].

BMI and smoking are genetically correlated traits [16]. Among the IVs for smoking initiation, 5% (15/297) are overlapping or highly correlated (*r*^2^ ≥ 0.8) with the IVs for BMI. Hence, we performed a multivariable MR (MVMR) to estimate the independent effects of smoking initiation and BMI on MS risk. The IVW linear regression model with multiplicative random effects was used for multivariable MR.

## Results

### Smoking phenotypes not associated with MS risk

**Fig. 1:**
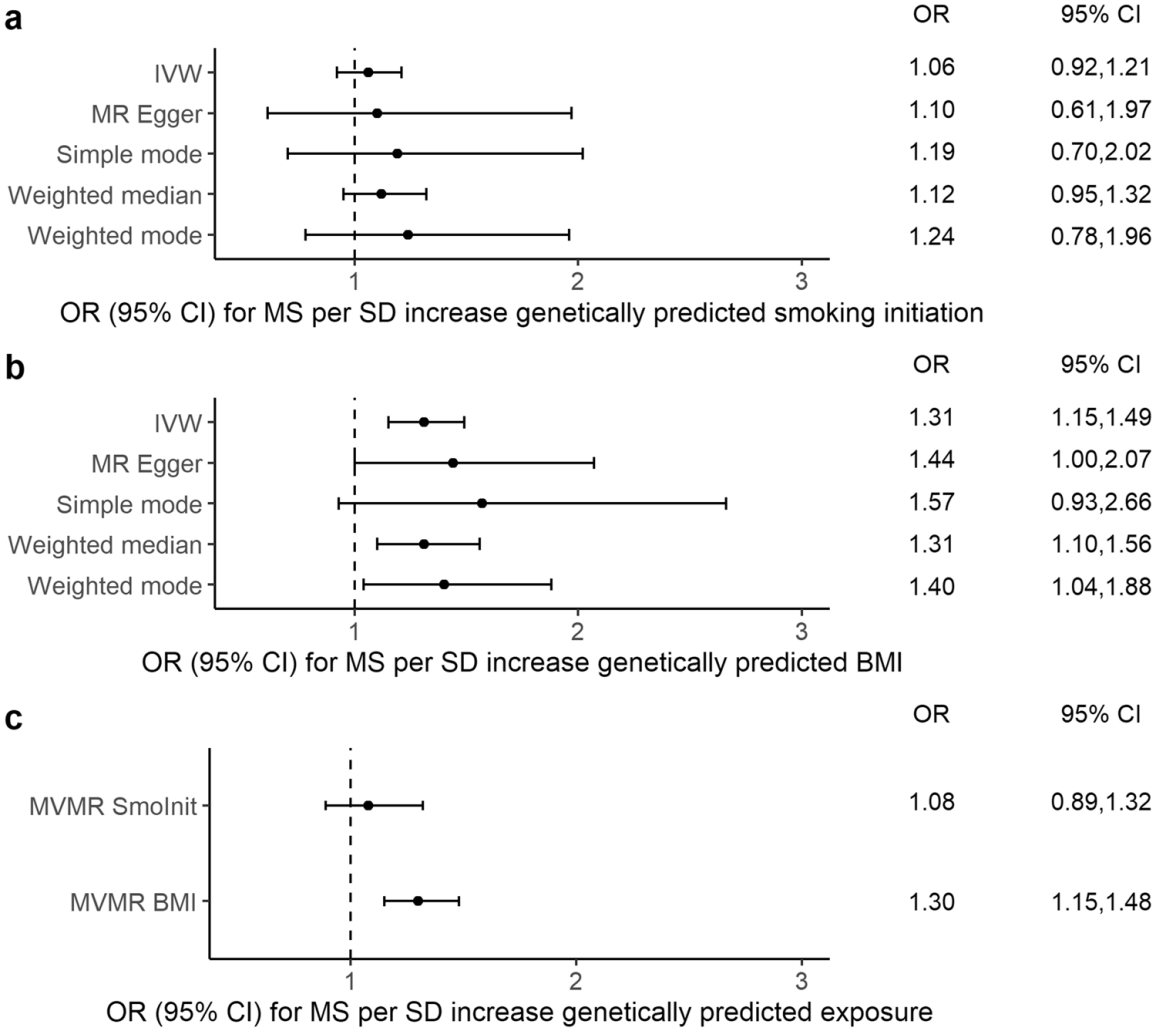
Mendelian randomization (MR) estimates of smoking initiation and BMI with multiple sclerosis (MS) from the primary analysis (IVW) and sensitivity analyses Data are displayed as odds ratio (OR) and 95% confidence interval (CI) per SD increment in (**a**) genetically predicted smoking initiation (**b**) genetically predicted BMI (**c**) genetically predicted smoking initiation and BMI, respectively. *IVW* inverse-variance weighted method, *MVMR* multivariable Mendelian randomization. In the original GWASs, a SD increase in genetically predicted smoking initiation and BMI corresponds to a 10% increased risk of smoking and an increase of 4.7 kg/m^2^, respectively [16, 18]

In an MR analysis, genetic predisposition to smoking initiation was not associated with MS risk using the random-effects inverse variance weighted (IVW) method [OR: 1.06, 95% confidence interval (CI) 0.92, 1.21, *p* = 0.42], and findings from the sensitivity tests were consistent (Fig. 1a). The scatter plot of the individual SNP estimates of smoking initiation and MS risk is shown in Supplementary Fig. 4a. The Cochran Q test and *I*^2^ statistic revealed moderate heterogeneity among the individual SNP effect estimates in the IVW analysis (*Q* = 443, *p* = 6.12 × 10^−8^; *I*^2^ = 33%). There was no evidence for directional pleiotropy from the MR Egger regression intercept [Egger intercept − 0.0008, 95% CI (− 0.012, 0.010), *p* = 0.89] and no asymmetry in the funnel plot (Fig. 3a).

**Fig. 2:**
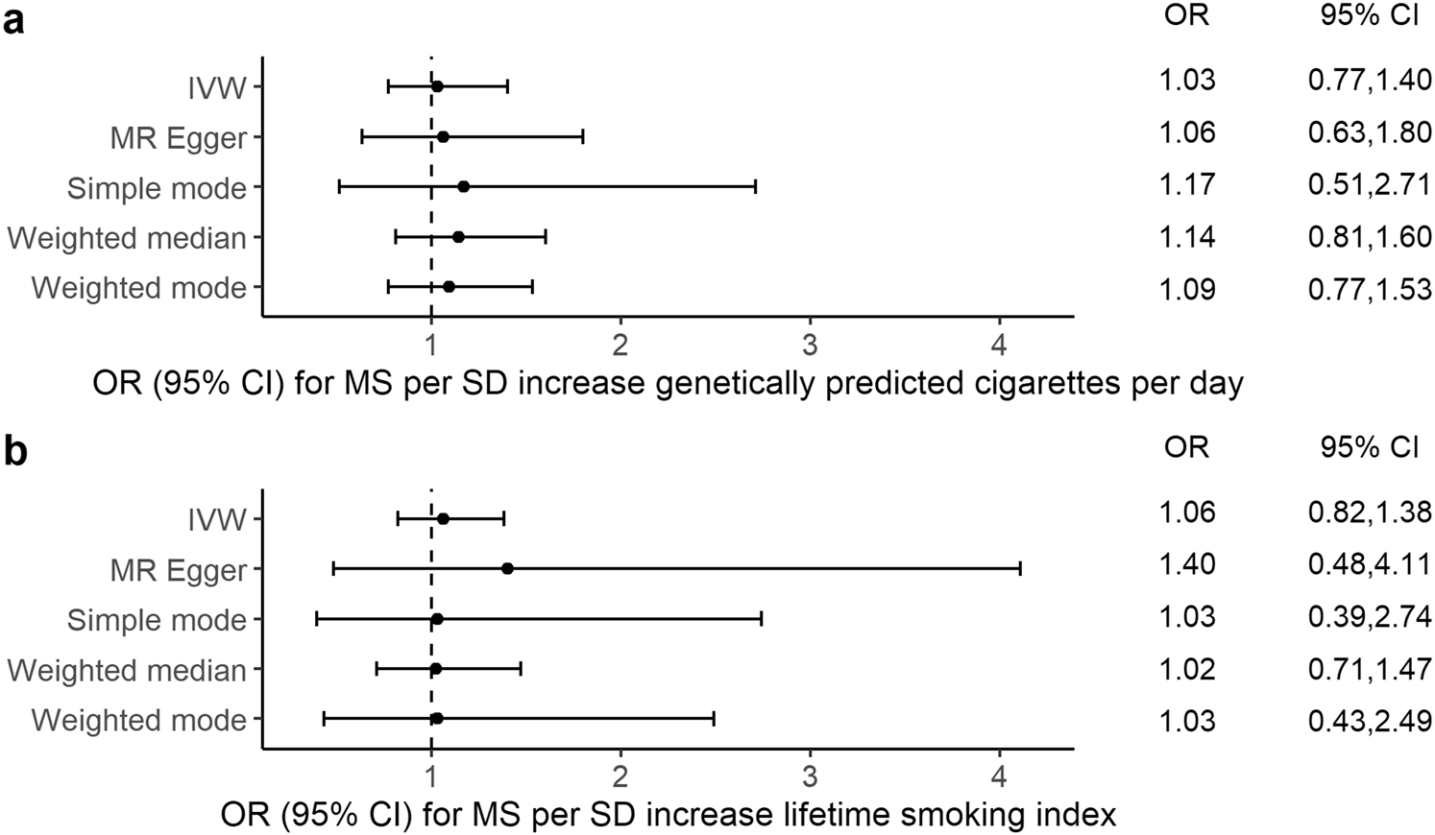
Mendelian randomization (MR) estimates of cigarettes per day and the lifetime smoking index with multiple sclerosis (MS) from the primary analysis (IVW) and sensitivity analyses Data are displayed as odds ratio (OR) and 95% confidence interval (CI) per SD increment in (**a**) genetically predicted cigarettes per day (**b**) lifetime smoking index. *IVW* inverse-variance weighted method. In the original GWASs, a SD increase in genetically predicted cigarettes per day corresponds to three additional daily cigarettes [16]

**Fig. 3:**
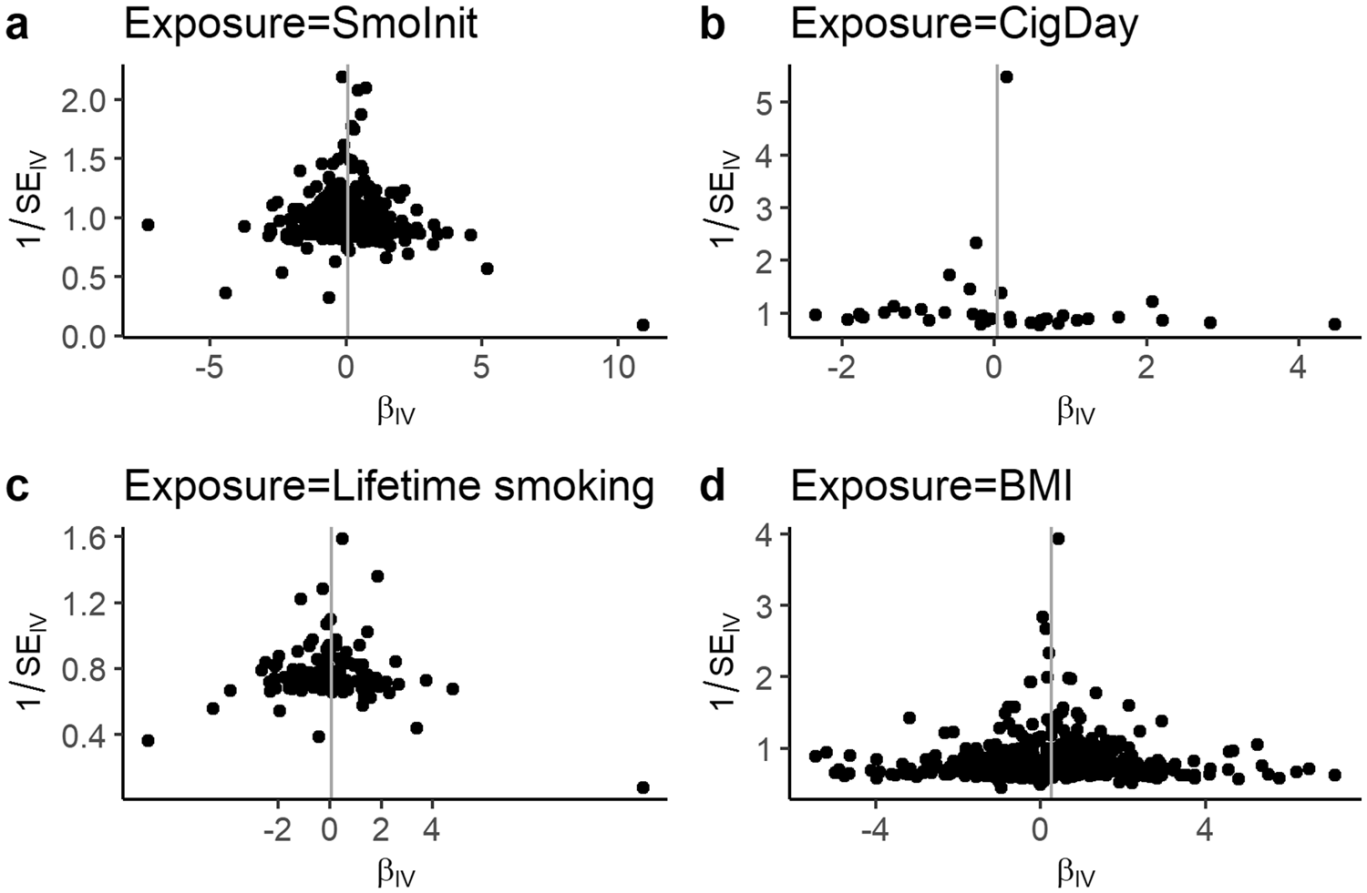
Funnel plots for the effect of smoking phenotypes and BMI on risk of MS For each single-nucleotide polymorphism (SNP), the resulting Mendelian randomization (MR) estimate is plotted against the inverse of the standard error of the MR estimate. Symmetry noted in this plot provides evidence against the presence of directional horizontal pleiotropy. The vertical line represents the summary measure of the effect of (**a**) smoking initiation (**b**) cigarettes per day (**c**) lifetime smoking index (**d**) BMI on risk of multiple sclerosis (MS) on the log-odds ratio scale

Genetically predicted smoking heaviness, expressed as cigarettes per day, was similarly not associated with MS risk in the main analysis [OR: 1.03, 95% CI (0.77, 1.40), *p* = 0.83] nor in sensitivity tests (Fig. 2a, Supplementary Fig. 4b). The individual SNP effect estimates showed moderate heterogeneity (*Q* = 61, *p* = 0.007, *I*^2^ = 40%). MR-Egger regression analyses suggested that pleiotropy did not greatly influence the results of the MR analyses [Egger intercept − 0.0008, 95% CI (− 0.0143, 0.0126), *p* = 0.90], and neither did the funnel plot suggest directional pleiotropy (Fig. 3b).

Integrating different IVs into a measure reflecting lifetime smoking was not associated with MS risk in the main analysis [OR: 1.06, 95% CI (0.82, 1.38), *p* = 0.65] nor in sensitivity tests (Fig. 2b, Supplementary Fig. 4c). The individual SNP effect estimates showed only limited heterogeneity (*Q* = 138, *p* = 0.04, *I*^2^ = 21%). There was no evidence for directional pleiotropy from the MR Egger regression intercept [Egger intercept − 0.0042, 95% CI (− 0.0201, 0.0112), *p* = 0.60] (Fig. 3c).

MR provides the possibility to explore reverse causation, a concern in observational studies. In our data, genetically predicted MS risk was not associated with smoking initiation [OR = 1.00, 95% CI (0.99, 1.01), *p* = 0.96], or lifetime smoking [*β* = 0.002, 95% CI (− 0.003, 0.007), *p* = 0.51], with a possible trend seen only for smoking heaviness [*β* = 0.016, 95% CI (0.003, 0.028), *p* = 0.02].

### Smoking phenotypes and BMI are correlated

As smoking and BMI are genetically correlated [16], we evaluated their relation in an MR approach. The IVW analysis of BMI and smoking initiation was highly significant and appeared bidirectional. Each genetically determined SD increase in BMI was associated with an increased likelihood of smoking initiation [OR: 1.21, 95% CI (1.17, 1.26), *p* = 8.64 × 10^−21^] (Supplementary Figs. 1a, 5a). Reversely, genetic predisposition to smoking was positively associated with BMI [*β* = 0.16, 95% CI (0.11, 0.21), *p* = 1.68 × 10^−11^] (Supplementary Figs. 1b, 5c). All sensitivity tests supported these findings (Supplementary Fig. 1), and there was no evidence for directional pleiotropy [Egger intercept − 0.0002, 95% CI (− 0.0020, 0.0016), *p* = 0.85 and Egger intercept − 0.0025, 95% CI (− 0.0061, 0.0011), *p* = 0.18] (Supplementary Fig. 2a, c). The individual SNP effect estimates did show substantial to considerable heterogeneity (*Q* = 1953, *p* = 5.29 × 10^−162^; *I*^2^ = 73% and *Q* = 4143, *p* < 1.0 × 10^−200^; *I*^2^ = 94%).

For smoking heaviness, MR effects were one-directional. Genetically predicted BMI was positively associated with the number of cigarettes per day [*β* = 0.35, 95% CI (0.31, 0.40), *p* = 3.60 × 10^−49^], consistent across all sensitivity analyses (Supplementary Figs. 3a, 5b). Genetically predicted smoking heaviness, on the other hand, was not correlated with BMI [*β* = 0.04, 95% CI [− 0.04, 0.12), *p* = 0.31] (Supplementary Figs. 3b, 5d). For the latter, there was, however, considerable heterogeneity (*Q* = 454, *p* = 1.88 × 10^−78^, *I*^2^ = 94%) and evidence for directional pleiotropy [Egger intercept 0.0065, 95% CI (0.0020, 0.0109), *p* = 0.0088] (Supplementary Fig. 2d), and sensitivity tests accounting for pleiotropy indicated even a possible inverse relation (Supplementary Fig. 3b).

### BMI associated with MS risk

Previously performed MR analyses have already demonstrated a causal positive association between BMI and MS risk [11, 13]. In our univariable analysis employing 529 BMI-associated SNPs as IVs to the largest MS genetics dataset, each genetically determined SD increase in adult BMI was associated with a 31% increased risk for MS [OR: 1.31, 95% CI (1.15, 1.49), *p* = 5.63 × 10^−5^] (Fig. 1b). The scatter plot of the individual SNP estimates of BMI and MS risk is shown in Supplementary Fig. 4d. The Cochran Q test and *I*^2^ statistic revealed moderate heterogeneity among the individual SNP effect estimates in the IVW analysis (*Q* = 947, *p* = 3.02 × 10^−26^, *I*^2^ = 44%). There was no evidence of significant unbalanced horizontal pleiotropy [Egger intercept − 0.0016, 95% CI (− 0.0075, 0.0042), *p* = 0.58], and neither did the funnel plot suggest directional pleiotropy (Fig. 3d), and all sensitivity tests provided similar estimates with overlapping confidence intervals (Fig. 1b).

### Multivariable MR: BMI but not smoking affects MS risk

A multivariable MR analysis allows to simultaneously adjust for the genetically predicted effects of two correlated exposures, smoking initiation and BMI, using the weighted regression-based framework. This multivariable analysis provided estimates for MS risk that were consistent with and nearly identical to the univariable results (Fig. 1c). BMI [OR: 1.30, 95% CI (1.15, 1.48), *p* = 2.78 × 10^−5^] but not smoking initiation [OR: 1.08, 95% CI (0.89, 1.32), *p* = 0.69] conferred an increased risk of MS.

## Discussion

In this MR study investigating the role of smoking in MS risk, we did not find evidence for causal effects of genetically predicted smoking initiation, smoking heaviness or lifetime smoking index on MS susceptibility.

For low serum vitamin D and increased BMI, MR analyses agree with observational studies and support a causal association with increased MS susceptibility [11–15]. For smoking, in contrast, our findings do not confirm the observational literature. Observational studies have repeatedly found an increased risk for MS in smokers versus non-smokers as described by subsequent meta-analyses [27–29]. They also observed that smoking correlates with MS risk in a dose-dependent manner, with both duration and intensity of smoking associated with an increased MS risk, and this effect is regardless of age at exposure [30]. No association was seen for conversion from clinically isolated syndrome (CIS) to MS [28]. The most recent meta-analysis finds little evidence for publication bias but reviews other limitations, which mostly do not reflect the quality of the studies but are inherent to the observational study design. Limitations listed are the retrospective case–control design that is prone to recall bias in a large majority of studies (89%), smoking status based on self-reporting which tends to lead to under-reporting in most studies (94%), and a low response/participation rate and/or a noticeably different response/participation rate between cases and controls in up to 25% of studies [28]. Despite overall evidence in observational studies for a dose–response relationship between smoking and MS risk, our MR study could not confirm a causal relation between genetically predicted smoking status (ever versus never smoker) or smoking heaviness (cigarettes smoked per day) and risk for MS.

MR studies are based on their own assumptions, which need to be checked adequately. Pleiotropy, that is an effect of a genetic variant on the outcome that is independent of the exposure, is an important concern. We addressed this by quantifying the MR Egger intercept as the average effect of IVs, independent of the exposure, by inspecting funnel plots and by applying four sensitivity analyses that are more robust to pleiotropy than the IVW analysis that is standard in MR. For all analyses of exposures on MS risk, there is no evidence of pleiotropy and results are consistent across the main analysis and sensitivity tests. Co-incident geographical variation in genotypes and health traits may bias genetic studies, including MR [31]. The GWAS studies that we used as basis for IVs only included individuals of European ancestry, and residual stratification was further corrected at the meta-analytic level with study-specific genomic controls. Moreover, latitude and ancestry were not associated with smoking in observational studies [29]. The use of the same study cohort to identify the genetic variants associated with an exposure and to apply them as IVs for association with an outcome may bias the MR results towards the exposure-outcome association from observational studies. For the relationship between BMI and smoking, the inclusion of UK Biobank results in a large overlap in individuals between the GSCAN and GIANT studies. Hence, the exact causal direction and effect size for BMI-smoking should be regarded with caution but we mainly wish to emphasize the correlation between the two traits as supported by different methods. The overlap in samples between exposures and MS is much less prominent, although it cannot be excluded or quantified precisely. Importantly, we observe a discrepancy with rather than a bias towards observational studies for the exposure of smoking.

A possible reason for the discrepancy between observational and MR studies could be weak instrument bias, where the strength of the known genetic IVs associated with the exposure is insufficient. For smoking initiation, however, IVs explain 2.3% of variance in the trait, and this is similar to the strength of 97 IVs (2.7%) for BMI in earlier, positive MR studies in MS [11, 13]. Also, in contrast to MS, other MR studies including smoking initiation as exposure variable were in line with observational studies for traits such as the risk for ischemic stroke [32], coronary artery disease [33] and type 2 diabetes [34], but also for neurological diseases such as amyotrophic lateral sclerosis [35] and autoimmune diseases such as rheumatoid arthritis [36]. Hence, although weak instrument bias cannot be excluded completely, other explanations for the discrepancy between observational and MR studies specifically in MS should be considered.

Two MS risk factors suggested by observational studies, that is smoking initiation/heaviness and BMI, are genetically correlated traits (*r* = 0.12–0.19) as demonstrated by LD-score regression [16]. Using the most recent IVs and summary statistics for BMI and smoking phenotypes, we replicate earlier evidence for a bidirectional relationship between BMI and smoking initiation [37], and demonstrate a unidirectional relationship for BMI on smoking heaviness. Both in a univariable MR and a multivariable MR, each SD increase in BMI, corresponding to roughly 5 kg/m^2^ units, conferred a 30% increase in MS risk. This effect and its size are in line with earlier MR studies starting from 97 instead of the currently known 656 genetic variants as IVs [11, 13]. Our MR study indicates that smoking does not have a direct effect on MS risk nor explains part of the association between BMI and MS risk. This is in line with the absence of an overall genetic correlation between smoking and MS using LD-score regression [16]. The observational studies associating smoking with MS susceptibility show heterogeneity in adjusting for established risk factors and other sociodemographic factors [27–29]. The vast majority of studies (86%) accounted for age and/or gender but only 6% of studies controlled for potential confounding by BMI [28], which we have here demonstrated to be highly correlated with both smoking and MS risk. Any residual confounding by this and other factors should hence be excluded.

Although MR provides an elegant approach to the study of causal relationships for exposures, it has limitations in capturing specific aspects of such relationships which may assist in understanding the discrepancies between observational studies and MR. First, the risk of smoking on MS in observational studies seems time-dependent, as it is higher for current versus past smoking [38], and abates 5 years after smoking cessation [39]. MR, on the other hand, typically captures the genetic predisposition to an exposure during one’s life-time, such as ever having smoked regularly. MR approaches that better capture time-varying exposures are now being developed [40]. The lifetime smoking score applied here is an example of such an approach. It captures well the known time- and dose-varying causal association of smoking with lung cancer (OR > 4) and cardiovascular diseases, and is correlated with known smoking-induced demethylation at the *AHRR* locus [17, 33]. Importantly, all IVs for smoking initiation, heaviness and lifetime smoking provide essentially identical results for MS with no evidence for an association. Secondly, the strength of the BMI IVs may differ by smoking status, as a 20% increase in the effect of the BMI genetic score on the actually observed BMI was seen in current smokers compared to former or never smokers [37]. Novel MR approaches such as factorial MR allow to model interactions between BMI and smoking phenotypes or an effect modification of smoking on BMI [41]. These studies necessitate the availability of individual-level genetic data, which is typically limited for large-scale studies.

Striking changes in the demographic pattern of multiple sclerosis (MS), with increasing prevalence and incidence over time, strongly indicate an influence of modifiable exposures on the disease [42]. Modifiable exposures lend themselves well to intervention, but it is important to pinpoint which of the many environmental, lifestyle, and sociodemographic changes that have occurred over the past decades such as higher smoking and obesity rates are responsible. MR is an elegant tool to leverage human genetics to inform prevention strategies in MS [43]. Despite observational studies repeatedly reporting an association between smoking and MS, our MR analyses on smoking phenotypes and MS risk could not confirm a causal relationship. The reasons for the discrepancy between observational studies and our MR study require further investigation, along the lines discussed. In addition, it remains to be determined whether interventions such as smoking cessation after disease onset, when the patient comes to the attention of the neurologist, do have an effect. Factors shaping the disease course after onset appear to be different from factors triggering disease [44, 45], implying that dedicated observational and MR studies are required to investigate factors underlying disease evolution [43, 46].

## Electronic supplementary material

Below is the link to the electronic supplementary material.

Supplementary file1 (DOCX 347 kb)

Supplementary file2 (XLSX 359 kb)

## Data Availability

This study makes use of summary statistics of genome-wide association studies that are publicly available (for smoking and BMI) or that have been shared by the International Multiple Sclerosis Genetics Consortium (for MS), as described in detail under the heading Genetic Datasets in the Methods section.
