# Supplementary material for: Smoking and multiple sclerosis risk: a Mendelian randomization study
Source: J Neurol. 2020 Jun 11;267(10):3083–91. doi: 10.1007/s00415-020-09980-4 (PMC7501136; doi:10.1007/s00415-020-09980-4)
Supplement: Supplementary file 1 — Supplementary file1 (DOCX 347 kb) [file 415_2020_9980_MOESM1_ESM.docx]

**Smoking and multiple sclerosis risk: A Mendelian randomization study**

Marijne Vandebergh^1,2^, An Goris^1,2^

^1^ KU Leuven - Department of Neurosciences, Laboratory for Neuroimmunology, Belgium

^2^ Leuven Brain Institute, KU Leuven, Belgium

Corresponding author:

Prof. An Goris

Laboratory for Neuroimmunology, Department of Neurosciences, KU Leuven

Herestraat 49 bus 1022, 3000 Leuven, Belgium

Phone: +32-16-330772

Email: an.goris@kuleuven.be

**Electronic supplementary material**

| **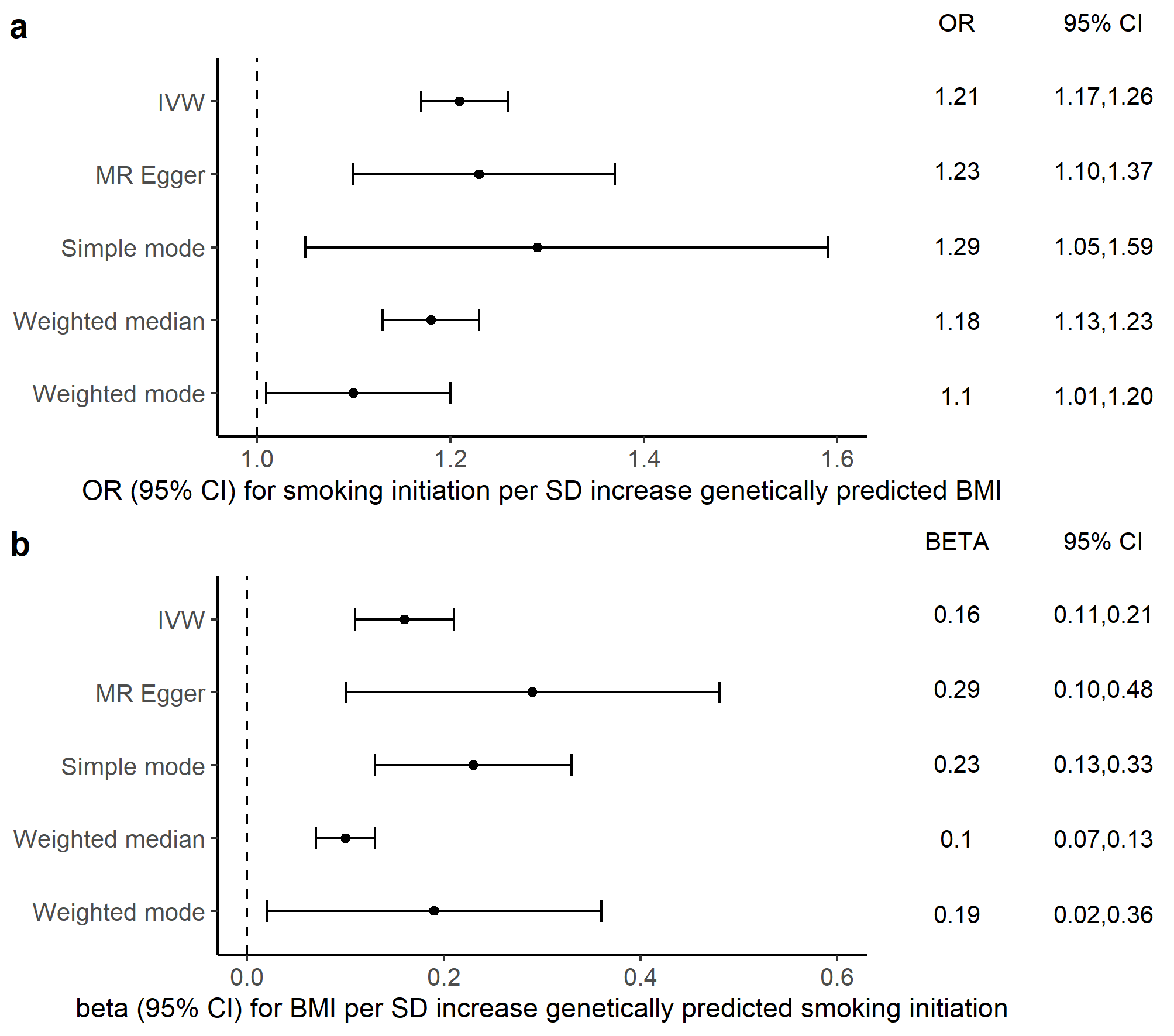** |
| --- |

**Supplementary Figure 1 Mendelian randomization (MR) estimates of BMI with smoking initiation and reversely, from the primary analysis (IVW) and sensitivity analyses** (a) Data are displayed as odds ratio (OR) and 95% confidence interval (CI) per SD increment genetically predicted BMI (b) Data are displayed as beta per SD increase genetically predicted smoking initiation
IVW= inverse-variance weighted method. In the original GWASs, a SD increase in genetically predicted smoking initiation and BMI corresponds to a 10% increased risk of smoking and an increase of 4.7 kg/m^2^, respectively [1,2].

| 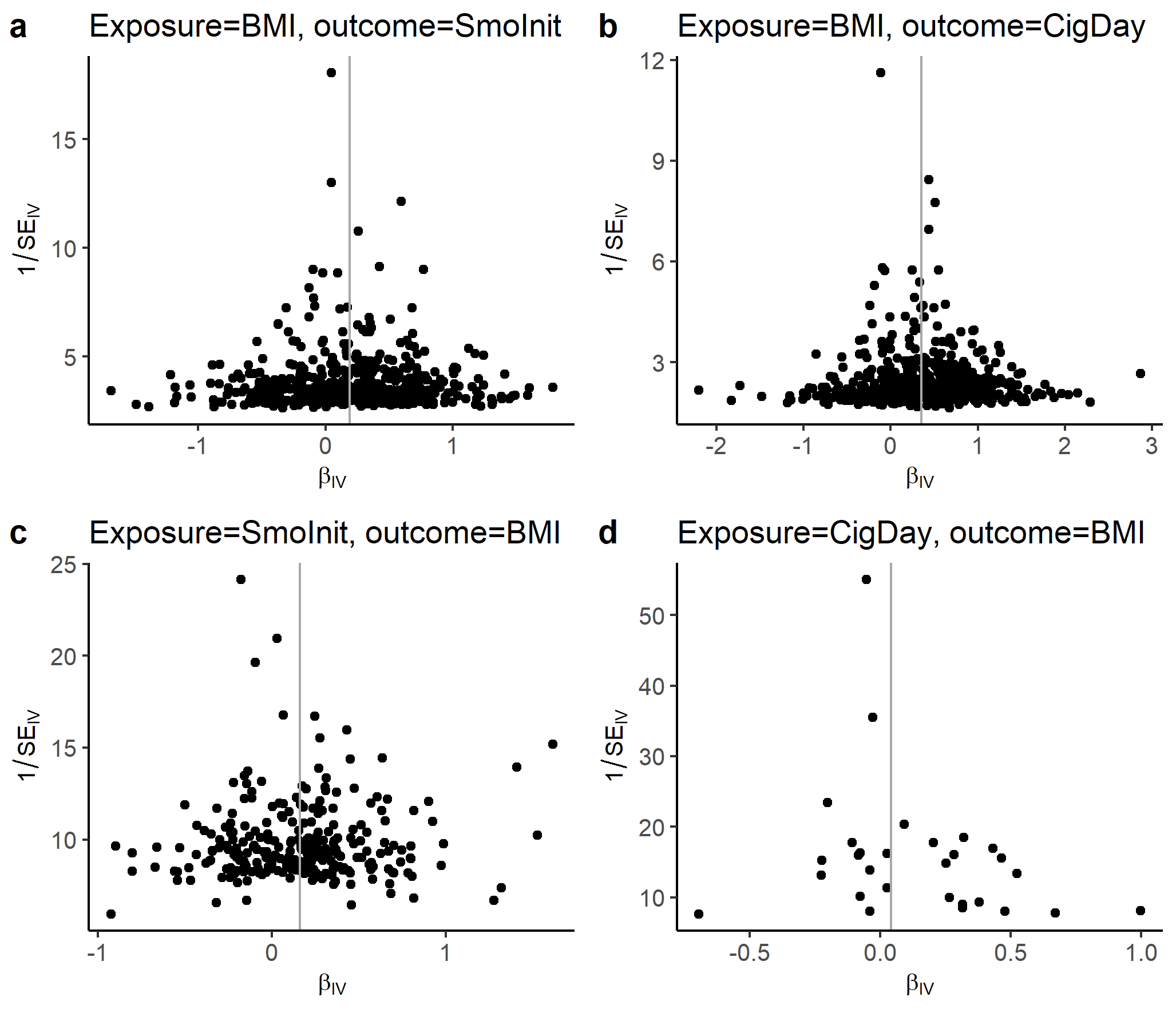 |
| --- |

**Supplementary Figure 2 Funnel plots for the effect of smoking phenotypes on BMI and reversely** For each single-nucleotide polymorphism (SNP), the resulting mendelian randomization (MR) estimate is plotted against the inverse of the standard error of the MR estimate. Symmetry noted in this plot provides evidence against the presence of directional horizontal pleiotropy. (a) The vertical line represents the summary measure of the effect of (a) BMI on smoking initiation (b) BMI on cigarettes per day (c) smoking initiation on BMI (d) cigarettes per day on BMI on the log-odds ratio scale.

| **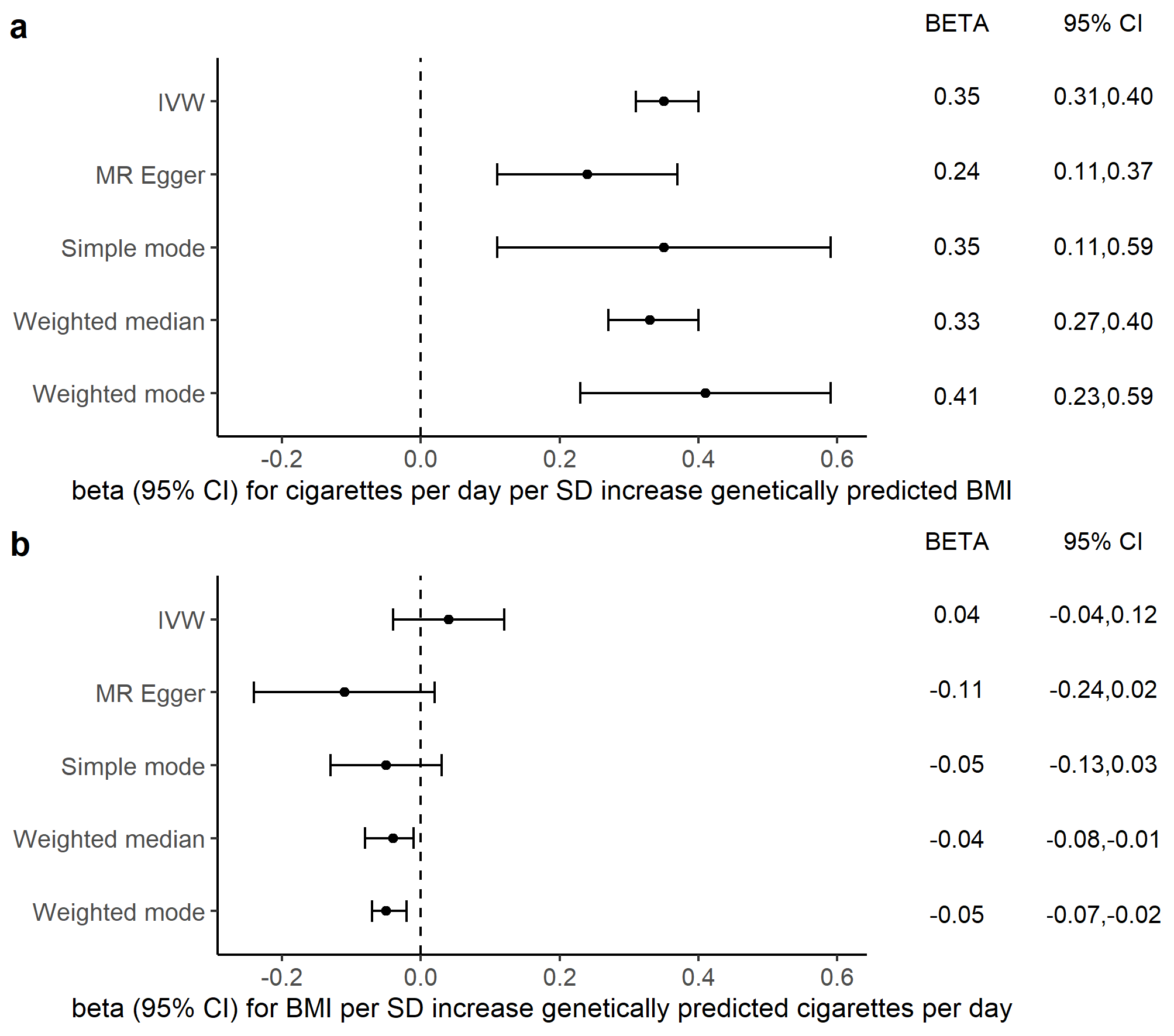** |
| --- |

**Supplementary Figure 3 Mendelian randomization (MR) estimates of BMI with cigarettes per day and reversely, from the primary analysis (IVW) and sensitivity analyses** (a) Data are displayed as beta and 95% confidence interval (CI) (a) per SD increment genetically predicted BMI (b) per SD increase genetically predicted cigarettes per day, IVW= inverse-variance weighted method. In the original GWASs, a SD increase in genetically predicted smoking heaviness and BMI corresponds to 3 additional daily cigarettes and an increase of 4.7 kg/m^2^, respectively [1,2].

| 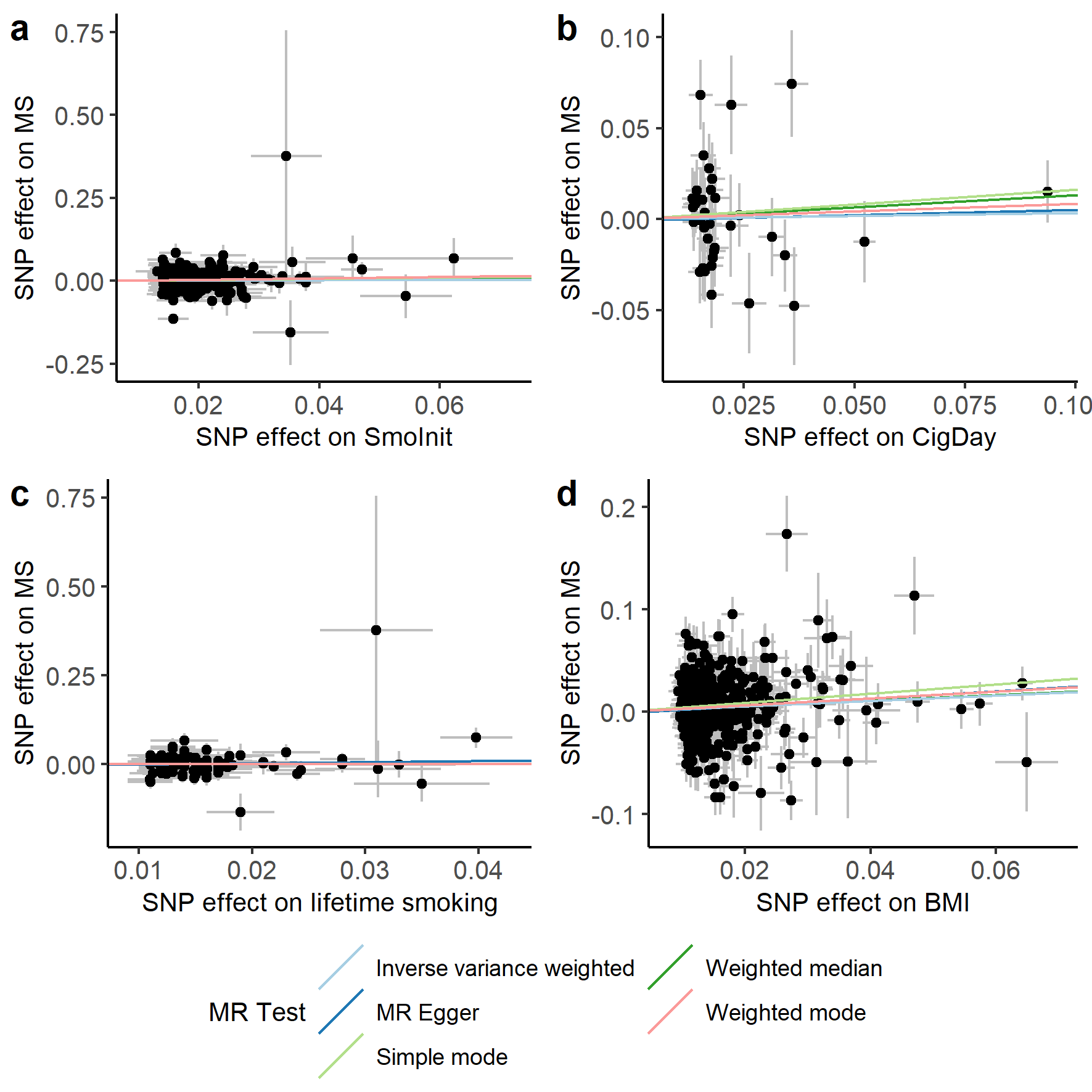 |
| --- |

**Supplementary Figure 4 Scatter plots of individual SNP estimates of exposures smoking initiation, cigarettes per day, lifetime smoking index and BMI and outcome MS risk** MR exposure variables are depicted on the x-axis, outcome variables on the y-axis.

| 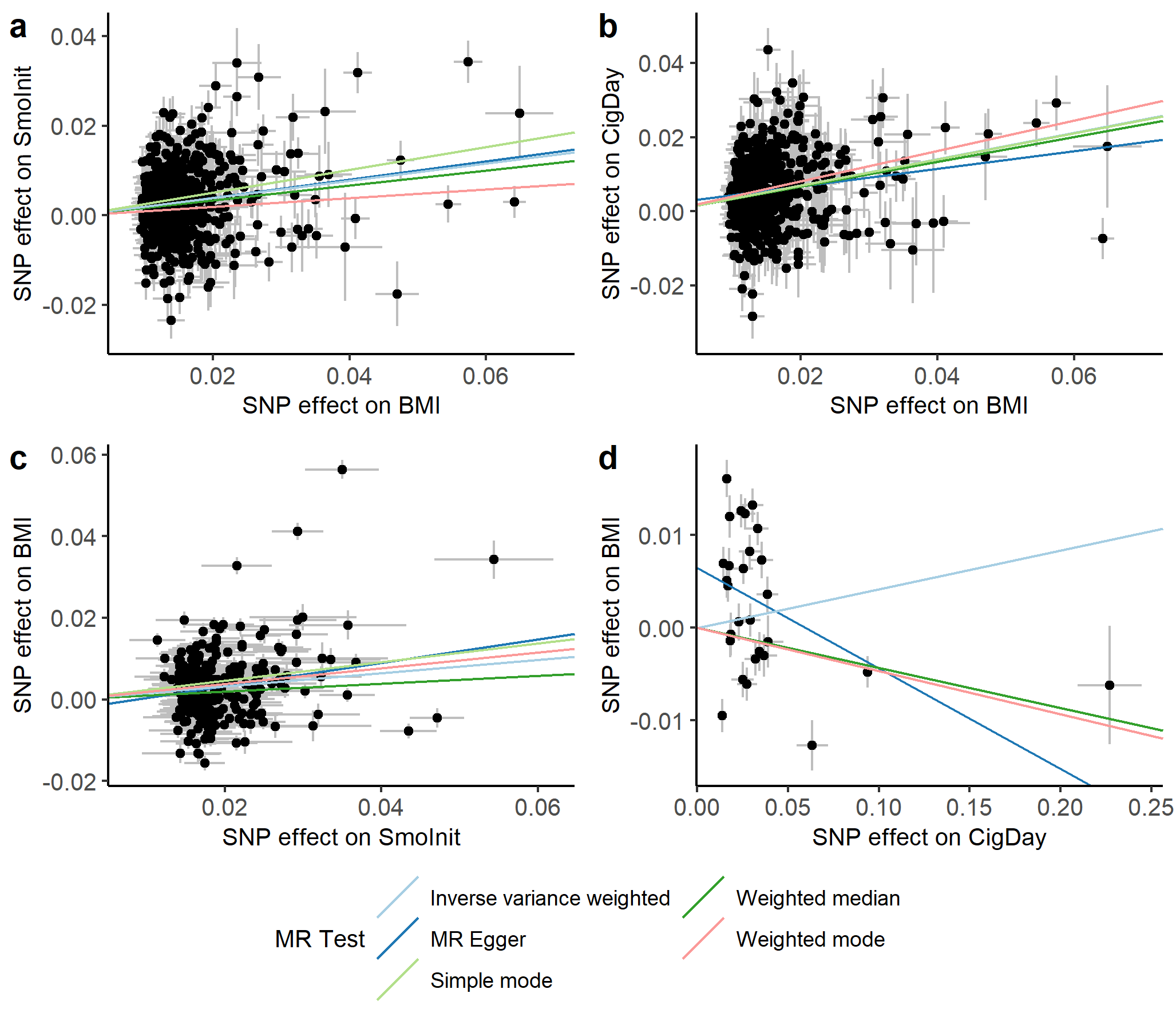 |
| --- |

**Supplementary Figure 5 Scatter plots of individual SNP estimates for MR between smoking initiation, cigarettes per day and BMI** MR exposure variables are depicted on the x-axis, outcome variables on the y-axis.

**References**

1. Liu M, Jiang Y, Wedow R, Li Y, Brazel DM, Chen F, Datta G, Davila-Velderrain J, McGuire D, Tian C, Zhan X, andMe Research T, Psychiatry HA-I, Choquet H, Docherty AR, Faul JD, Foerster JR, Fritsche LG, Gabrielsen ME, Gordon SD, Haessler J, Hottenga JJ, Huang H, Jang SK, Jansen PR, Ling Y, Magi R, Matoba N, McMahon G, Mulas A, Orru V, Palviainen T, Pandit A, Reginsson GW, Skogholt AH, Smith JA, Taylor AE, Turman C, Willemsen G, Young H, Young KA, Zajac GJM, Zhao W, Zhou W, Bjornsdottir G, Boardman JD, Boehnke M, Boomsma DI, Chen C, Cucca F, Davies GE, Eaton CB, Ehringer MA, Esko T, Fiorillo E, Gillespie NA, Gudbjartsson DF, Haller T, Harris KM, Heath AC, Hewitt JK, Hickie IB, Hokanson JE, Hopfer CJ, Hunter DJ, Iacono WG, Johnson EO, Kamatani Y, Kardia SLR, Keller MC, Kellis M, Kooperberg C, Kraft P, Krauter KS, Laakso M, Lind PA, Loukola A, Lutz SM, Madden PAF, Martin NG, McGue M, McQueen MB, Medland SE, Metspalu A, Mohlke KL, Nielsen JB, Okada Y, Peters U, Polderman TJC, Posthuma D, Reiner AP, Rice JP, Rimm E, Rose RJ, Runarsdottir V, Stallings MC, Stancakova A, Stefansson H, Thai KK, Tindle HA, Tyrfingsson T, Wall TL, Weir DR, Weisner C, Whitfield JB, Winsvold BS, Yin J, Zuccolo L, Bierut LJ, Hveem K, Lee JJ, Munafo MR, Saccone NL, Willer CJ, Cornelis MC, David SP, Hinds DA, Jorgenson E, Kaprio J, Stitzel JA, Stefansson K, Thorgeirsson TE, Abecasis G, Liu DJ, Vrieze S (2019) Association studies of up to 1.2 million individuals yield new insights into the genetic etiology of tobacco and alcohol use. Nat Genet 51 (2):237-244. doi:10.1038/s41588-018-0307-5

2. Yengo L, Sidorenko J, Kemper KE, Zheng Z, Wood AR, Weedon MN, Frayling TM, Hirschhorn J, Yang J, Visscher PM, Consortium G (2018) Meta-analysis of genome-wide association studies for height and body mass index in approximately 700000 individuals of European ancestry. Hum Mol Genet 27 (20):3641-3649. doi:10.1093/hmg/ddy271
